# Black Soybean and Adzuki Bean Extracts Lower Blood Pressure by Modulating the Renin-Angiotensin System in Spontaneously Hypertensive Rats

**DOI:** 10.3390/foods10071571

**Published:** 2021-07-06

**Authors:** Eun-Woo Jeong, Se-Yeong Park, Yun-Sun Yang, You-Jin Baek, Da-Min Yun, Hyun-Joo Kim, Gwang-Woong Go, Hyeon-Gyu Lee

**Affiliations:** 1 Department of Food and Nutrition, Hanyang University, Seoul 04763, Korea; bravoadria@hanyang.ac.kr (E.-W.J.); psydkwk@hanyang.ac.kr (S.-Y.P.); diddbstjs777@hanyang.ac.kr (Y.-S.Y.); jyyj161126@hanyang.ac.kr (Y.-J.B.); fdamin@hanyang.ac.kr (D.-M.Y.); 2 Department of Central Area Crop Science, National Institute of Crop Science, Wanju-Gun 55365, Korea; tlrtod@korea.kr; 3 Korean Living Science Research Center, Hanyang University, Seoul 04763, Korea

**Keywords:** angiotensin-converting enzyme, black soybean, adzuki bean, blood pressure, spontaneously hypertensive rat

## Abstract

Hypertension, causing cardiovascular disease, stroke, and heart failure, has been a rising health issue worldwide. Black soybeans and adzuki beans have been widely consumed throughout history due to various bioactive components. We evaluated the antihypertensive effects of black soybean and adzuki bean ethanol extracts on blood pressure, renin-angiotensin system (RAS), and aortic lesion in spontaneously hypertensive rats. A group of WKY (normal) and six groups of spontaneously hypertensive rats were administered with saline (SHR), 50 mg/kg of captopril (CAP), 250 and 500 mg/kg of black soybean extracts (BE250 and BE500), 250 and 500 mg/kg of adzuki bean extracts (AE250 and AE500) for eight weeks. BE250, BE500, AE250, and AE500 significantly (*p* < 0.05) reduced relative liver weight, AST, ALT, triglyceride, total cholesterol, systolic blood pressure, and angiotensin-converting-enzyme level compared to SHR. The angiotensin II level in AE500 and renin mRNA expression in BE500 and AE500 were significantly (*p* < 0.05) decreased compared to SHR. The lumen diameter was significantly (*p* < 0.05) reduced in only CAP. Furthermore, systolic and diastolic blood pressure and angiotensin II level in AE500 were lower than those of BE500. These results suggest that AE exhibit more antihypertensive potential than BE in spontaneously hypertensive rats.

## 1. Introduction

Hypertension, defined as increased systolic blood pressure (≥140 mmHg) and/or diastolic blood pressure (≥90 mmHg) (WHO), is a global health issue [1,2]. The prevalence of hypertension was estimated at 1.13 billion adults in 2015 [3]; such prevalence is consistently rising. Underlying this epidemic is the rapidly aging population and indiscriminate eating habits [4]. Accordingly, the worldwide socio-economic burden of hypertension was estimated at 81 billion dollars, including medical expenses and economic losses caused by productivity decline [5]. More importantly, the rising prevalence of hypertension increases mortality from conditions such as cardiovascular disease, chronic kidney failure, and dementia. As such, hypertension threatens public health, consequently degrading the quality of individual life [6].

Various antihypertensive medicines, such as angiotensin-converting enzyme inhibitors, angiotensin receptor blockers, calcium channel blockers, and beta-blockers, have been discovered. Nonetheless, hypertension remains largely uncontrolled [7]. The antihypertensive drugs could have undesirable side effects, including respiratory tract abstraction, angioedema, dyspnea, cough, hair loss, and headache [8]. Therefore, dietary supplements are preferred as alternatives to drugs in that they have fewer side effects and are easy to absorb. The antihypertensive effects of various natural extracts have been verified in vitro and in vivo [9,10,11]. These activities are associated with their flavonoids, terpenes, alkaloids, and phenolic acids [12]. Indeed, several dietary supplements are on the market that lower blood pressure with the rapid growth of the global functional food market [13].

Black soybeans (*Glycine max*) and adzuki beans (*Vigna angularis*) are commonly consumed worldwide. Both are well known as effective antioxidant foods because of their high phenolic compounds. Bai et al. reported that black soybean extracts using 95% ethanol contains isoflavones (genistein, daidzein, 2′-hydroxydaidzein, daidzin, glycitin, genistin, acetyldaidzin, acetylglycitin, and acetylgenistin), flavones (isoquescitrin), phenolic acids (chlorogenic acid), aurantiamide acetate, and phaseic acid, etc. [14]. Besides, Lee et al. reported adzuki bean extracts using 80% ethanol includes gallic acid, catechin, ferulic acid, and hesperidin, etc. [15]. Accordingly, various functionalities of black soybeans and adzuki beans have been reported. The ethanolic extracts of black soybeans, for instance, exerted notable antioxidant, anti-inflammatory, antinociceptive, and antiplatelet activities [16,17,18]. In addition, adzuki bean ethanolic extracts attenuated angiogenesis, diabetes, osteoporosis, muscle atrophy, and allergic inflammation, as well as delay in the progression of Alzheimer’s disease [19,20,21,22,23]. Having integrated that, black soybeans and adzuki beans have the potential to be applied as functional foods. A few studies conducted the antihypertensive activity of adzuki bean extracts in aspects of macrophage infiltration, vascular oxidative stress, inflammation, blood pressure elevation, and NO production [24,25,26]. However, none of the previous studies have elucidated the antihypertensive activities of black soybeans or adzuki beans regarding the renin-angiotensin system (RAS). Therefore, we hypothesize that the black soybean extracts and adzuki bean extracts would alleviate high blood pressure and regulate the significant components of the RAS in the spontaneously hypertensive rats (SHR).

## 2. Materials and Methods

### 2.1. Sample Preparation

The black soybeans (*Glycine max*, Chungja #5) and adzuki beans (*Vigna angularis*, Arari) cultivars harvested in 2019 were provided by the National Institute of Crop Science (Rural Development Administration, Suwon, Korea). The legumes were pulverized using a grinder and stirred using 100% ethanol (100 g/1 L) for 24 h at room temperature. The extracts were filtered using Whatman No.2 filter paper and evaporated using a rotary vacuum evaporator (Eyela, Tokyo, Japan) at 50 °C. The black soybean extracts (BE) and adzuki bean extracts (AE) were stored at −80 °C until further experiments.

### 2.2. Animals and Diets

All experiments were approved by the Animal Ethics Committee of Woojung Bio (WJIACUC20200326-1-41). Six-week-old male Wistar-Kyoto rats (WKY) and spontaneously hypertensive rats (SHR) were obtained from Central Lab (Animal Ltd., Seoul, Korea). All rats were housed in a controlled environment (22 ± 2 °C, 45 ± 5% humidity, and 12 h light cycle). Food (SAFE A40, SAFE Incorporation, Augy, France) and water were available *ad libitum*. After a week of acclimation, animals were assigned into seven groups (*n* = 6): (1) WKY (saline), a normal control group, (2) SHR (saline), a negative control group, (3) CAP (50 mg/kg body weight (bw) of captopril), a positive control group, (4) BE250 (250 mg/kg bw of black soybean extracts), (5) BE500 (500 mg/kg bw of black soybean extracts), (6) AE250 (250 mg/kg bw of adzuki bean extracts), and (7) AE500 (500 mg/kg bw of adzuki bean extracts).

There was no previous in vivo study evaluating the antihypertensive effect of black soybean extracts. In the previous studies to evaluate the antihypertensive effect of adzuki bean extracts, adzuki bean extracts were fed with feed rather than oral gavage [24,25]. Consequently, the experimental doses (250 and 500 mg/kg) of BE and AE were determined based on previous studies related to other physiological activities rather than antihypertensive activity [27,28]. All treatments were dissolved in 0.5% (*w*/*v*) carboxymethyl cellulose in saline (Sigma-Aldrich, St. Louis, MO, USA), and orally administered to the rats every day for eight weeks.

### 2.3. Growth Performance and Organ Weights

Body weight and feed intake were measured once a week throughout the experiment. After eight weeks, the rats were fasted for 12 h and anesthetized with 2% isoflurane (2 L/min). The liver, heart, and aorta were immediately excised, weighed, and stored at −80 °C until further analysis.

### 2.4. Blood Pressure Measurement

Systolic and diastolic blood pressure were measured using the non-invasive tail-cuff method with a LE 5002 (Panlab Inc., Barcelona, Spain) once a week. In brief, unanesthetized rats were placed in a chamber preheated at 37 °C. The cuff was placed on the tail and connected to a compressed air cylinder inflated and deflated at a constant rate. Blood pressure was measured in triplicate.

### 2.5. Blood Biochemical Analysis

Blood was collected from the caudal vena cava and divided into two tubes for serum and plasma. Serum aspartate aminotransferase (AST) and alanine transaminase (ALT) were measured using commercial kits (Asan Pharmaceutical, Seoul, Korea). The levels of plasma triglyceride (TG), total cholesterol (TC), and low-density lipoprotein cholesterol (LDL-C) were analyzed by using an autoanalyzer (Hitachi, Tokyo, Japan). The angiotensin-converting enzyme (ACE) level (Cusabio Corporation, Wuhan, China) and angiotensin II level in serum (Raybiotech Inc., Norcross, GA, USA) were measured using the rat ELISA kit.

### 2.6. Real-Time PCR

The kidney tissue was homogenized using TRIzol (Ambion, Austin, TX, USA). The total RNA was separated using TRIzol/chloroform, precipitated with isopropanol, and washed using ethyl alcohol in order. The NanoDrop (Thermo Fisher Scientific, Waltham, MA, USA) was used to determine RNA quantity and quality. The concentration and purification of RNA were estimated by measuring the absorbance at 260/280 and 260/230 nm using spectrophotometry. Purified RNA of each sample was used for cDNA synthesis using a Prime Script™ RT reagent kit (Takara, Shiga, Japan). PCR amplification was performed with SYBR green to detect relative mRNA expression using the CFX96TM RT-PCR detection system (Bio-Rad, Hercules, CA, USA). The primer sequences for PCR were as follows; renin (NM_012642.4) forward primer, 5′-TGCTAAAGGAGGAAGTGTTT-3′; renin reverse primer, 5′-TGATGCTCACGTAGTGAAAG-3′; GAPDH (NM_017008.4) forward primer, 5′-GTCGGTGTGAACGGATTTG-3′, GAPDH reverse primer, 5′-TCCCATTCTCAGCCTTGAC-3′. Each value was normalized to GAPDH, and the relative expression levels of the genes were calculated using the delta-delta threshold cycle (ΔΔCt) method compared to the SHR.

### 2.7. Histological Analysis of Aorta

The aorta of rats was fixed in 4% formaldehyde and embedded in paraffin. Samples were stained with hematoxylin and eosin, and stained areas were viewed through a microscope at 20× magnification. The aorta’s lumen diameter and media thickness were measured by Image J software (National Institutes of Health, Bethesda, MD, USA).

### 2.8. Statistical Analysis

Data are shown as mean ± standard error of the mean (SEM). The results were analyzed by a one-way analysis of variance (one-way ANOVA), followed by Tukey’s post hoc test using GraphPad Prism 8 (GraphPad Software, La Jolla, CA, USA). The differences were considered statistically significant if *p* < 0.05.

## 3. Results and Discussion

### 3.1. Growth Performance and Organ Weights

Growth performance and organ weights of WKY and SHR groups are presented in Table 1. The body weight of SHR was significantly lowered compared to WKY (*p* < 0.05). This change was not due to a reduction of feed intake; conversely, the feed intake of SHR was even higher than that of WKY (*p* < 0.05). These observations were consistent with the previous studies that SHR showed lower weight and higher feed intake than WKY [26,29,30]. The theory that explains this phenomenon is that SHR, having a high concentration of angiotensin II, evokes the sympathetic nervous system’s excitation and causes lower weight [31]. In our study, BE and AE did not change either the weight or feed intake. According to the previous findings, black soybean pulse powder did not alter body weight and feed intake in SHR [32], and adzuki bean extracts using 80% ethanol also did not change body weight in SHR [24,25].

The liver weight was increased in SHR compared to WKY (*p* < 0.05), which was rescued in CAP, indicating orderly model induction. Similarly, a previous study demonstrated consistent observation [33]. The liver weight of all treatments, including BE250, BE500, AE250, and AE500, was lowered compared to SHR (*p* < 0.05). The relationship between high blood pressure and liver functions has not yet been established. Some studies, nonetheless, reported an increased risk of liver injury in patients with high blood pressure, and vice versa [34,35,36]. From this perspective, the reduction and recovery of liver weight by BE and AE observed in this study are recognized as a positive indicator of liver function recovery.

More interestingly, there is compelling evidence that BE and AE modulate hepatic lipid metabolism. According to a previous study, black soybean seed coat extracts reduced the liver weight by polyphenols, including anthocyanin and procyanidin, preventing visceral fat accumulation by activating AMPK, increasing hepatic β-oxidation, and inhibiting de novo lipogenesis in KKAy mice [37]. Accordingly, adzuki bean seed coat extracts 1.0% mixed with feed decreased relative liver weight in SHR [26]. Adzuki bean powder ameliorated hepatic lipogenesis by inhibiting SREBP-1c and FAS mRNA expression and increased hepatic β-oxidation by increasing PPARα and CPT-1 mRNA expression with a decrement of liver weight in non-alcoholic fatty liver disease mice [38].

In sum, orderly induction of the SHR model could be affirmed in the current study; however, no alteration of body weight and feed intake by BE or AE was observed. The reduction of liver weight by BE and AE has provided supplemental findings for improving liver function and hepatic lipid accumulation despite the fact that no in-depth research was conducted due to the scope.

### 3.2. Blood Biochemical Parameters

The serum levels of hepatotoxicity index were shown in Table 2. Following a previous study, the levels of aspartate transaminase (AST) and alanine aminotransferase (ALT) in SHR were higher than those of WKY (*p* < 0.05) despite still in normal ranges [33]. CAP, BE250, BE500, AE250, and AE500 decreased AST and ALT levels compared to SHR (*p* < 0.05). Similarly, black soybean powder decreased AST in the high cholesterol/fat diet-induced nonalcoholic fatty liver disease mice model [39]. The ethanol extracts from adzuki beans significantly decreased AST and ALT in high fat diet-induced obese mice [40]. In addition, adzuki bean water extracts reduced serum AST in a rat model that showed hepatotoxicity by acetaminophen [41]. Therefore, BE and AE up to 500 mg/kg have the potential to improve the biomarkers of liver function.

Plasma lipid profiles were examined to identify the role of black soybean and adzuki bean extracts in lipid homeostasis (Table 2). The plasma levels of triglyceride (TG) and total cholesterol (TC) in SHR were higher than those of WKY (*p* < 0.05). CAP, BE250, BE500, AE250, and AE500 reduced TG and TC levels compared to SHR (*p* < 0.05). More interestingly, CAP, BE500, AE250, and AE500 decreased LDL-C levels compared to SHR (*p* < 0.05). It was previously reported that 60% ethanol extracts of black soybeans improved TC, LDL-C, and HDL-C in overweight and obese adults in a randomized, double-blinded clinical trial [42]. Likewise, black soybeans processed by various methods, including boiling, freeze-drying, and coarse-milling, decreased TC and LDL-C levels in SHR [43]. Besides, a 40% ethanol fraction of hot-water extracts of adzuki beans improves hepatic lipid profiles with a reduction of TG (12%) and TC (7%) in the KKAy mice model [44]. Taken together, BE and AE presented the possibility of relieving the biomarkers of dyslipidemia and cardiovascular diseases in SHR.

### 3.3. Systolic Blood Pressure and Diastolic Blood Pressure

**Figure 1:**
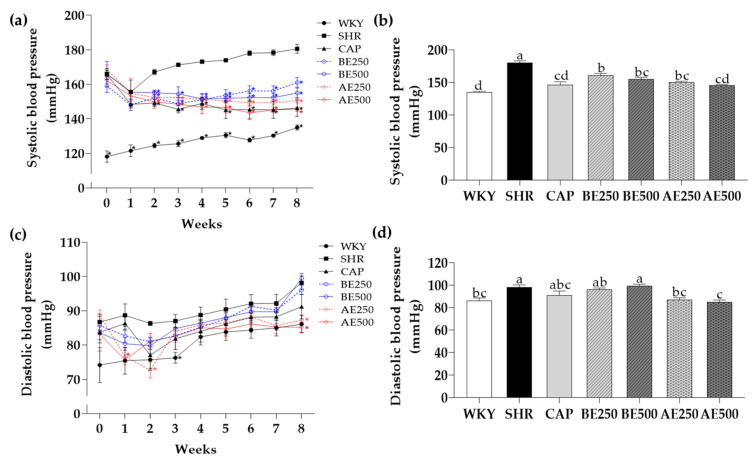
(**a**) Weekly systolic blood pressure, (**b**) systolic blood pressure at week 8, (**c**) weekly diastolic blood pressure, and (**d**) diastolic blood pressure at week 8 in the rats administered with black soybean or adzuki bean extracts. Data are expressed as mean ± SEM. * indicates a significant difference when compared to SHR group. The values with different letters indicate significant differences at *p* < 0.05. WKY: Wistar-Kyoto rats, SHR: Spontaneously hypertensive rats, CAP: 50 mg/kg body weight (bw) of captopril, BE250: 250 mg/kg bw of black soybean extracts, BE500: 500 mg/kg bw of black soybean extracts, AE250: 250 mg/kg bw of adzuki bean extracts, AE500: 500 mg/kg bw of adzuki bean extracts.

The systolic blood pressure (SBP) of SHR, the decisive risk factor of hypertension and cardiovascular disease, was significantly (*p* < 0.05) higher than WKY throughout the experiment (Figure 1). These results indicate that the SHR model was well established as a hypertensive model. Interestingly, the SBP of CAP, BE250, BE500, AE250, and AE500 has dramatically decreased compared to SHR since the 2nd week (*p* < 0.05). At the end of treatment, BE250, BE500 (11% and 14%), AE250, and AE500 (17% and 19%) showed lower SBP in a dose-dependent manner. Even the degree of blood pressure reduction caused by BE500, AE250, and AE500 was similar to that of the positive control group, CAP.

These findings agreed with a previous study in which 0.8% or 0.9% adzuki bean extracts mixed with feed lowered SBP in SHR [24,25]. Adzuki bean extracts up-regulated NO production via stimulating eNOS and iNOS in the aorta and kidney, resulting in reduced SBP. On the other hand, no studies have been reported yet to specify the efficacy of black soybean extracts on SBP. Instead, a study that provided healthy women with daily black soybeans for 4 weeks has reported indirect evidence such as reduced oxidative stress, followed by improved blood vessel function [45]. In addition, supplementation of mixed legumes, including lentils, chickpeas, and peas, to obese humans, reduced the SBP compared to the control [46].

Concerning the diastolic blood pressure (DBP), AE500 showed lower DBP compared to SHR in the 1st week and AE250 decreased DBP in the 2nd week. AE250 and AE500 decreased DBP than SHR in the final week (11% and 13%, respectively; *p* < 0.05). The DBP reduced by AE250 and AE500 is nearly equivalent to that of WKY. The decrease of SBP by BE and AE and the decline of DBP by AE suggested that BE and AE, especially AE, could be possible substances to alleviate hypertension.

### 3.4. Angiotensin-Converting Enzyme and Angiotensin Ⅱ Level in Serum, and Renin mRNA Expression in the Kidney

**Figure 2:**
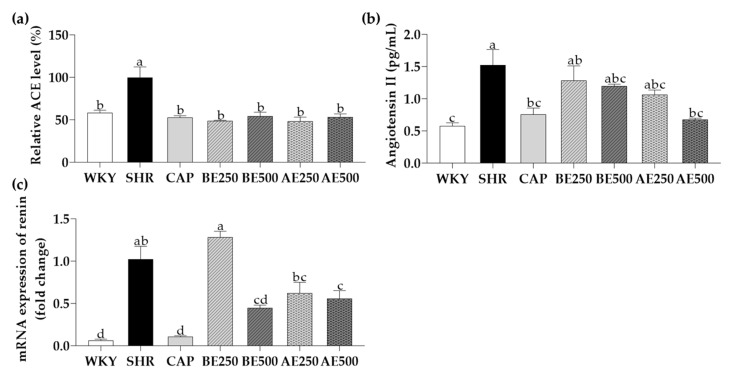
Effects of black soybean or adzuki bean extracts on (**a**) relative angiotensin converting enzyme (ACE) level (%), (**b**) angiotensin II level in serum, and (**c**) renin mRNA expression in renal tissue of the rats administered with black soybean or adzuki bean extracts for 8 weeks. Data are expressed as mean ± SEM. The values with different letters indicate significant differences at *p* < 0.05. WKY: Wistar-Kyoto rats, SHR: Spontaneously hypertensive rats, CAP: 50 mg/kg body weight (bw) of captopril, BE250: 250 mg/kg bw of black soybean extracts, BE500: 500 mg/kg bw of black soybean extracts, AE250: 250 mg/kg bw of adzuki bean extracts, AE500: 500 mg/kg bw of adzuki bean extracts.

The renin-angiotensin system (RAS) is a key axis for regulating blood pressure and is a major target for hypertension drugs and nutraceuticals. Renin, a major enzyme of the RAS, can cleave angiotensinogen to form angiotensin I, which is further converted to vasoconstrictor angiotensin II by ACE in the lung. ACE is a zinc metallopeptidase activated by chloride and plays a pivotal role in regulating blood pressure. ACE inhibitors, such as captopril, bind to the active site competing with angiotensin I. Angiotensin II raises blood pressure by a series of actions; it stimulates the sympathetic nervous system, increases aldosterone biosynthesis, and produces vasoconstriction and renal actions. Therefore, in the current study, we analyze ACE and angiotensin II levels and renin mRNA expression to validate the effects of BE and AE on the RAS (Figure 2).

As leading results of successful experimental models validated earlier, the relative ACE level (%) in serum was significantly higher in SHR than in WKY. CAP, BE250, BE500, AE250, and AE500 exhibited significantly lower ACE levels in serum than SHR (*p* < 0.05). Notable differences in the ACE levels among BE250, BE500, AE250, and AE500 were not observed. Although the level of an enzyme does not entirely reveal an enzyme activity, previous studies and manufacturer’s protocols suggest that the enzyme ACE level in serum is sufficiently representative of its activity. In fact, our findings are associated with the previous results, in which black soybeans showed the highest in vitro ACE inhibitory activity than black turtle beans and lentils [47]. Similarly, adzuki bean extracts showed in vitro ACE inhibitory activity, similar to captopril (0.25 µg/mL) [48]. As a source of such functionality of black soybean extracts and adzuki bean extracts, flavonoids, alkaloids, and tannins in extracts play a role in hydrogen bonding at the active site of ACE as a competitive inhibitor, form chelate complexes with the zinc, or precipitate protein [49,50].

The angiotensin II level was significantly lower in SHR compared to WKY. There was reduced angiotensin II in AE500 compared to SHR (*p* < 0.05), which accorded with the SBP and DBP as validated earlier. The mRNA expression of renin in renal tissue was significantly suppressed in BE500 (56%) and AE500 (45%) compared to SHR (*p* < 0.05). Likewise, a previous study has affirmed that adzuki bean water extracts exerted the highest renin inhibitory activity among little legumes [51]. Collectively, AE could improve hypertension by modulating ACE, angiotensin II, and renin.

### 3.5. Histological Findings of the Aorta

**Figure 3:**
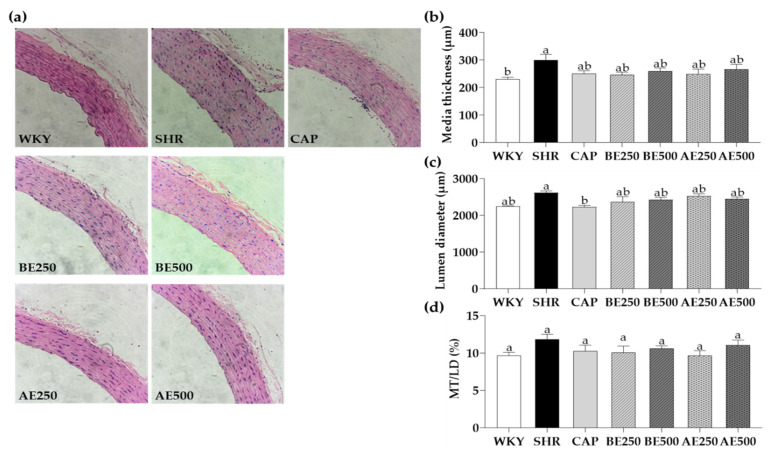
Histological findings with (**a**) H&E staining of aorta, (**b**) media thickness (MT), (**c**) lumen diameter (LD), and (**d**) MT/LD of aorta in the rat administered with black soybean or adzuki bean extracts for 8 weeks. Data are expressed as mean ± SEM. The values with different letters indicate significant differences at *p* < 0.05. WKY: Wistar-Kyoto rats, SHR: Spontaneously hypertensive rats, CAP: 50 mg/kg body weight (bw) of captopril, BE250: 250 mg/kg bw of black soybean extracts, BE500: 500 mg/kg bw of black soybean extracts, AE250: 250 mg/kg bw of adzuki bean extracts, AE500: 500 mg/kg bw of adzuki bean extracts.

The effects of BE and AE on vascular remodeling in the aorta were evaluated using H&E staining (Figure 3). Media thickness (MT) is an index and independent marker of preclinical atherosclerosis and is negatively impacted by hypertension. MT of SHR (300.6 ± 20 µm) was greater compared to WKY (231.5 ± 6.2 µm); however, there was no influence on MT alteration generated by BE or AE. The lumen diameter (LD) of CAP was smaller than that of SHR (*p* < 0.05); otherwise, there was no difference in LD and the ratio of MT/LD among all groups.

There have been no previous studies to clarify the effects of BE and AE on the medium thickness and luminous diameter. However, some studies demonstrated the addressed factors through other indirect markers. For instance, Yao et al. reported that lentil extracts improved angiotensin II-induced vascular remodeling [52]. Mukai and Sato found that adzuki bean seed coats alleviated vascular oxidative stress produced by vascular smooth muscle cells in SHR by suppressing NADPH oxidase, the most important producer of O_2_^−^, composed of Nox4, p22phox, and p47phox [26]. In sum, our study did not exhibit the remarkable alteration of media thickness and lumen diameter; however, there was a marginal reduction of both markers. Further in-depth research is required to clarify the effect of BE and AE on vascular remodeling.

## 4. Conclusions

In conclusion, the current work demonstrated that oral administration of black soybean extracts and adzuki bean extracts in SHR remarkably rescued systolic blood pressure. The diastolic blood pressure was reduced in only AE250 and AE500. BE and AE lowered ACE level and renin mRNA expression. Furthermore, 500 mg/kg of AE showed predominant inhibition of the RAS system in SHR through suppressing ACE and angiotensin II levels and renin mRNA expression. These results suggest that both BE and AE have the potential as an anti-hypertensive nutraceutical by regulating RAS in SHR, and AE especially has shown stronger evidence than BE.

## Figures and Tables

**Table 1:** Growth performance and organ weights of the rats administered with black soybean or adzuki bean extracts for 8 weeks. WKY: Wistar-Kyoto rats, SHR: Spontaneously hypertensive rats, CAP: 50 mg/kg body weight (bw) of captopril, BE250: 250 mg/kg bw of black soybean extracts, BE500: 500 mg/kg bw of black soybean extracts, AE250: 250 mg/kg bw of adzuki bean extracts, AE500: 500 mg/kg bw of adzuki bean extracts.

	WKY	SHR	CAP	BE250	BE500	AE250	AE500
Body weight (g)	377 ± 2.73 ^a^	339 ± 3.41 ^bc^	351 ± 1.83 ^b^	342 ± 3.90 ^bc^	347 ± 4.32 ^bc^	341 ± 1.48 ^bc^	333 ± 4.32 ^c^
Feed intake (g/day)	23.9 ± 0.24 ^b^	25.5 ± 0.31 ^a^	25.5 ± 0.34 ^a^	24.7 ± 0.56 ^ab^	24.8 ± 0.40 ^ab^	25.1 ± 0.29 ^ab^	24.6 ± 0.14 ^ab^
Liver weight (g)	9.69 ± 0.36 ^d^	18.2 ± 0.32 ^a^	12.5 ± 0.54 ^b^	11.0 ± 0.15 ^cd^	11.9 ± 0.15 ^bc^	12.0 ± 0.10 ^cd^	11.0 ± 0.33 ^bc^
Relative liver weight (%)	2.57 ± 0.09 ^c^	5.19 ± 0.11 ^a^	3.79 ± 0.01 ^b^	3.23 ± 0.01 ^b^	3.49 ± 0.08 ^b^	3.39 ± 0.06 ^b^	3.37 ± 0.18 ^b^
Heart weight (g)	1.29 ± 0.04 ^b^	1.75 ± 0.04 ^a^	1.63 ± 0.01 ^a^	1.63 ± 0.10 ^a^	1.72 ± 0.08 ^a^	1.65 ± 0.02 ^a^	1.78 ± 0.03 ^a^
Relative heart weight (%)	0.35 ± 0.01 ^b^	0.51 ± 0.00 ^a^	0.47 ± 0.00 ^a^	0.47 ± 0.03 ^a^	0.48 ± 0.02 ^a^	0.49 ± 0.01 ^a^	0.51 ± 0.01 ^a^

Data are expressed as mean ± SEM. The values with different letters in the same row indicate significant differences at *p* < 0.05.

**Table 2:** Blood biochemical parameters in serum and plasma of the rats administered with black soybean or adzuki bean extracts for 8 weeks. AST: aspartate transaminase, ALT: alanine aminotransferase, TG: triglyceride, TC: total cholesterol, LDL-C: Low density lipoprotein cholesterol.

	WKY	SHR	CAP	BE250	BE500	AE250	AE500
AST (IU/L)	18.6 ± 1.87 ^b^	24.7 ± 1.95 ^a^	16.5 ± 0.65 ^bc^	14.4 ± 0.69 ^bcd^	11.2 ± 0.37 ^cd^	10.7 ± 0.72 ^cd^	10.0 ± 1.31^d^
ALT (IU/L)	4.29 ± 0.76 ^c^	19.3 ± 2.50 ^a^	8.05 ± 1.17 ^b c^	12.2 ± 1.46 ^b^	7.47 ± 0.60 ^bc^	7.36 ± 2.32 ^bc^	8.31 ± 0.82 ^bc^
TG (mg/dL)	37.6 ± 3.99 ^c^	135 ± 7.24 ^a^	74.5 ± 6.86 ^b^	83.4 ± 1.58 ^b^	97.2 ± 9.27 ^b^	74.2± 4.88 ^b^	74.9 ± 3.38 ^b^
TC (mg/dL)	88.4 ± 2.18 ^b^	95.6 ± 1.21 ^a^	77.7 ± 0.89 ^c^	79.2 ± 0.86 ^c^	78.8 ± 1.95 ^c^	75.8 ± 0.25 ^c^	80.3 ± 2.31 ^c^
LDL-C (mg/dL)	8.95 ± 0.26 ^ab^	9.53 ± 0.44 ^a^	7.73 ± 0.16 ^b^	8.88 ± 0.36 ^ab^	7.78 ± 0.34 ^b^	7.70 ± 0.07 ^b^	7.80 ± 0.18 ^b^

Data are expressed as mean ± SEM. The values with different letters in the same row indicate significant differences at *p* < 0.05.

## Data Availability

The data presented in this study are available on request from the corresponding author.

## References

[1] World Health Organization Factsheet on Hypertension. https://www.who.int/news-room/fact-sheets/detail/hypertension.

[2] Unger T., Borghi C., Charchar F., Khan N.A., Poulter N.R., Prabhakaran D., Ramirez A., Schlaich M., Stergiou G.S., Tomaszewski M. (2020). 2020 International society of hypertension global hypertension practice guidelines. Hypertension.

[3] Zhou B., Bentham J., Di Cesare M., Bixby H., Danaei G., Cowan M.J., Paciorek C.J., Singh G., Hajifathalian K., Bennett J.E. (2017). Worldwide trends in blood pressure from 1975 to 2015: A pooled analysis of 1479 population-based measurement studies with 19· 1 million participants. Lancet.

[4] Mills K.T., Bundy J.D., Kelly T.N., Reed J.E., Kearney P.M., Reynolds K., Chen J., He J. (2016). Global disparities of hypertension prevalence and control: A systematic analysis of population-based studies from 90 countries. Circulation.

[5] Wierzejska E., Giernaś B., Lipiak A., Karasiewicz M., Cofta M., Staszewski R. (2020). A global perspective on the costs of hypertension: A systematic review. Arch. Med. Sci..

[6] Burnier M., Egan B.M. (2019). Adherence in hypertension: A review of prevalence, risk factors, impact, and management. Circ. Res..

[7] Chobanian A.V. (2009). The hypertension paradox—more uncontrolled disease despite improved therapy. N. Engl. J. Med..

[8] Özkaya E., Yazganoğlu K.D. (2014). Adverse Cutaneous Drug Reactions to Cardiovascular Drugs.

[9] Dos Santos R.L., Dellacqua L.O., Delgado N.T., Rouver W.N., Podratz P.L., Lima L.C., Piccin M.P., Meyrelles S.S., Mauad H., Graceli J.B. (2016). Pomegranate peel extract attenuates oxidative stress by decreasing coronary angiotensin-converting enzyme (ACE) activity in hypertensive female rats. J. Toxicol. Environ. Health A.

[10] Tong R.-C., Qi M., Yang Q.-M., Li P.-F., Wang D.-D., Lan J.-P., Wang Z.-T., Yang L. (2019). Extract of *Plantago asiatica L.* seeds ameliorates hypertension in spontaneously hypertensive rats by inhibition of angiotensin converting enzyme. Front. Pharmacol..

[11] Balasuriya N., Rupasinghe H.V. (2012). Antihypertensive properties of flavonoid-rich apple peel extract. Food Chem..

[12] Maione F., Cicala C., Musciacco G., De Feo V., Amat A.G., Ialenti A., Mascolo N. (2013). Phenols, alkaloids and terpenes from medicinal plants with antihypertensive and vasorelaxant activities. A review of natural products as leads to potential therapeutic agents. Nat. Prod. Commun..

[13] Sachdeva V., Roy A., Bharadvaja N. (2020). Current prospects of nutraceuticals: A review. Curr. Pharm. Biotechnol..

[14] Bai Y., Xu Y., Wang B., Li S., Guo F., Hua H., Zhao Y., Yu Z. (2017). Comparison of phenolic compounds, antioxidant and antidiabetic activities between selected edible beans and their different growth periods leaves. J. Funct. Foods.

[15] Lee J.H., Ham H., Kim M.Y., Ko J.Y., Sim E.-Y., Kim H.-J., Lee C.K., Jeon Y.H., Jeong H.S., Woo K.S. (2018). Phenolic compounds and antioxidant activity of adzuki bean cultivars. Legume Res..

[16] Nur’aini F.D., Rahayu S., Rifa’i M. (2019). Anti-inflammatory activity of elicited soybean (*Glycine max*) extract on Balb/C mice (Mus musculus) with high-fat and-fructose diet. Cent. Eur. J. Immunol..

[17] Yim J.H., Lee O.-H., Choi U.-K., Kim Y.-C. (2009). Antinociceptive and anti-inflammatory effects of ethanolic extracts of *Glycine max (L.) Merr* and *Rhynchosia nulubilis* seeds. Int. J. Mol. Sci..

[18] Kim K., Lim K.-M., Kim C.-W., Shin H.-J., Seo D.-B., Lee S.-J., Noh J.-Y., Bae O.-N., Shin S., Chung J.-H. (2011). Black soybean extract can attenuate thrombosis through inhibition of collagen-induced platelet activation. J. Nutr. Biochem..

[19] Miyazaki H., Okamoto Y., Motoi A., Watanabe T., Katayama S., Kawahara S.-I., Makabe H., Fujii H., Yonekura S. (2019). Adzuki bean (*Vigna angularis*) extract reduces amyloid-β aggregation and delays cognitive impairment in drosophila models of Alzheimer’s disease. Nutr. Res. Pract..

[20] Kwon O.S., Jeong M.S., Kim B., Kim S.-H. (2015). Antiangiogenic effect of ethanol extract of *Vigna angularis* via inhibition of phosphorylation of VEGFR2, Erk, and Akt. J. Evid. Based Complementary Altern. Med..

[21] Sato S., Mukai Y., Kataoka S., Kurasaki M. (2016). Azuki bean (*Vigna angularis*) extract stimulates the phosphorylation of AMP-activated protein kinase in HepG2 cells and diabetic rat liver. J. Sci. Food Agric..

[22] Lim H.J., Park S.I., Bak S.G., Cheong S.H., Lee S., Baek Y.B., Lee C.M., Lee K.M., Lee S.W., Lee S.J. (2020). Beneficial effects of *Vigna angularis* extract in osteoporosis and osteoarthritis. Food Sci. Nutr..

[23] Kim C., Kim M.-B., Hwang J.-K. (2020). Red bean extract inhibits immobilization-induced muscle atrophy in C57BL/6N mice. J. Med. Food.

[24] Sato S., Mukai Y., Yamate J., Kato J., Kurasaki M., Hatai A., Sagai M. (2008). Effect of polyphenol-containing azuki bean (*Vigna angularis*) extract on blood pressure elevation and macrophage infiltration in the heart and kidney of spontaneously hypertensive rats. Clin. Exp. Pharmacol. Physiol..

[25] Mukai Y., Sato S. (2009). Polyphenol-containing azuki bean (*Vigna angularis*) extract attenuates blood pressure elevation and modulates nitric oxide synthase and caveolin-1 expressions in rats with hypertension. Nutr. Metab. Cardiovasc. Dis..

[26] Mukai Y., Sato S. (2011). Polyphenol-containing azuki bean (*Vigna angularis*) seed coats attenuate vascular oxidative stress and inflammation in spontaneously hypertensive rats. J. Nutr. Biochem..

[27] Chan K.-C., Kok K.-E., Huang K.-F., Weng Y.-L., Chung Y.-C. (2020). Effects of fermented red bean extract on nephropathy in streptozocin-induced diabetic rats. Food Nutr. Res..

[28] Park S.Y., Pak S., Kang S.J., Kim N.Y., Kim D.S., Kim M.J., Kim S.A., Kim J.Y., Park S.Y., Park S.H. (2015). Effects of the C3G/D3G anthocyanins-rich black soybean testa extracts on improvement of lipid profiles in STZ-induce diabetic rats. J. Nutr. Health.

[29] Kokubo M., Uemura A., Matsubara T., Murohara T. (2005). Noninvasive evaluation of the time course of change in cardiac function in spontaneously hypertensive rats by echocardiography. Hypertens. Res..

[30] Yang H.-Y., Yang S.-C., Chen J.-R., Tzeng Y.-H., Han B.-C. (2004). Soyabean protein hydrolysate prevents the development of hypertension in spontaneously hypertensive rats. Br. J. Nutr..

[31] Cabassi A., Vinci S., Cantoni A.M., Quartieri F., Moschini L., Cavazzini S., Cavatorta A., Borghetti A. (2002). Sympathetic activation in adipose tissue and skeletal muscle of hypertensive rats. Hypertension.

[32] Clark J.L., Loader T.B., Anderson H.D., Zahradka P., Taylor C.G. (2020). Regular black bean consumption is necessary to sustain improvements in small-artery vascular compliance in the spontaneously hypertensive rat. Nutrients.

[33] Fukuda S., Tsuchikura S., Iida H. (2004). Age-related changes in blood pressure, hematological values, concentrations of serum biochemical constituents and weights of organs in the SHR/Izm, SHRSP/Izm and WKY/Izm. Exp. Anim..

[34] Brookes M., Cooper B. (2007). Hypertension and fatty liver: Guilty by association?. J. Hum. Hypertens..

[35] Diehl A. (2004). Fatty liver, hypertension, and the metabolic syndrome. Gut.

[36] Stranges S., Trevisan M., Dorn J.M., Dmochowski J., Donahue R.P. (2005). Body fat distribution, liver enzymes, and risk of hypertension: Evidence from the Western New York Study. Hypertension.

[37] Kurimoto Y., Shibayama Y., Inoue S., Soga M., Takikawa M., Ito C., Nanba F., Yoshida T., Yamashita Y., Ashida H. (2013). Black soybean seed coat extract ameliorates hyperglycemia and insulin sensitivity via the activation of AMP-activated protein kinase in diabetic mice. J. Agric. Food Chem..

[38] Kim S., Hong J., Jeon R., Kim H.-S. (2016). Adzuki bean ameliorates hepatic lipogenesis and proinflammatory mediator expression in mice fed a high-cholesterol and high-fat diet to induce nonalcoholic fatty liver disease. Nutr. Res..

[39] Jung J.-H., Kim H.-S. (2013). The inhibitory effect of black soybean on hepatic cholesterol accumulation in high cholesterol and high fat diet-induced non-alcoholic fatty liver disease. Food Chem. Toxicol..

[40] Kim M., Pichiah P.B.T., Kim D.K., Cha Y.S. (2017). Black adzuki bean (*Vigna angularis*) extract exerts phenotypic effects on white adipose tissue and reverses liver steatosis in diet-induced obese mice. J. Food Biochem..

[41] Han K.-H., Fukushima M., Ohba K., Shimada K.-I., Sekikawa M., Chiji H., Lee C.-H., Nakano M. (2004). Hepatoprotective effects of the water extract from adzuki bean hulls on acetaminophen-induced damage in rat liver. J. Nuti. Sci. Vitaminol..

[42] Lee M., Sorn S.R., Park Y., Park H.-K. (2016). Anthocyanin rich-black soybean testa improved visceral fat and plasma lipid profiles in overweight/obese Korean adults: A randomized controlled trial. J. Med. Food.

[43] Loader T.B., Zahradka P., Ahmadi S., Taylor C.G. (2021). Processing method modulates the effectiveness of black beans for lowering blood cholesterol in spontaneously hypertensive rats. J. Sci. Food Agric..

[44] Itoh T., Kobayashi M., Horio F., Furuichi Y. (2009). Hypoglycemic effect of hot-water extract of adzuki (*Vigna angularis*) in spontaneously diabetic KK-Ay mice. Nutrition.

[45] Yamashita Y., Wang L., Nakamura A., Nanba F., Saito S., Toda T., Nakagawa J., Ashida H. (2020). Black soybean improves the vascular function through an increase in nitric oxide and a decrease in oxidative stress in healthy women. Arch. Biochem. Biophys..

[46] Hermsdorff H.H.M., Zulet M.Á., Abete I., Martínez J.A. (2011). A legume-based hypocaloric diet reduces proinflammatory status and improves metabolic features in overweight/obese subjects. Eur. J. Nutr..

[47] Zhang Y., Pechan T., Chang S.K. (2018). Antioxidant and angiotensin-I converting enzyme inhibitory activities of phenolic extracts and fractions derived from three phenolic-rich legume varieties. J. Funct. Foods.

[48] Yu M., Kim H.-J., Yu J., Lee H., Sung J., Jeong H.S., Lee J. (2020). Comparison of antioxidant and anti-hypertensive activities of ethanol extracts from cereal grains and legumes. J. Korean Soc. Food Sci. Nutr..

[49] Chen C.-H., Lin J.-Y., Lin C.-N., Hsu S.-Y. (1992). Inhibition of angiotensin-I-converting enzyme by tetrahydroxyxanthones isolated from *Tripterospermum lanceolatum*. J. Nat. Prod..

[50] Lacaille-Dubois M., Franck U., Wagner H. (2001). Search for potential angiotensin converting enzyme (ACE)-inhibitors from plants. Phytomedicine.

[51] Takahashi S., Hori K., Kumagai M., Wakabayashi S. (2007). Human renin inhibitory activity in legumes. ACE.

[52] Yao F., Sun C., Chang S.K. (2012). Lentil polyphenol extract prevents angiotensin II-induced hypertension, vascular remodelling and perivascular fibrosis. Food Funct..

